# The Roles of NRF2 in Modulating Cellular Iron Homeostasis

**DOI:** 10.1089/ars.2017.7176

**Published:** 2018-10-24

**Authors:** Michael John Kerins, Aikseng Ooi

**Affiliations:** Department of Pharmacology and Toxicology, College of Pharmacy, University of Arizona, Tucson, Arizona.

**Keywords:** NRF2, iron, oxygen, heme, cancer, ferroptosis

## Abstract

***Significance:*** Iron and oxygen are intimately linked: iron is an essential nutrient utilized as a cofactor in enzymes for oxygen transport, oxidative phosphorylation, and metabolite oxidation. However, excess labile iron facilitates the formation of oxygen-derived free radicals capable of damaging biomolecules. Therefore, biological utilization of iron is a tightly regulated process. The nuclear factor (erythroid-derived 2)-like 2 (NRF2) transcription factor, which can respond to oxidative and electrophilic stress, regulates several genes involved in iron metabolism.

***Recent Advances:*** The bulk of NRF2 transcription factor research has focused on its roles in detoxification and cancer prevention. Recent works have identified that several genes involved in heme synthesis, hemoglobin catabolism, iron storage, and iron export are under the control of NRF2. Constitutive NRF2 activation and subsequent deregulation of iron metabolism have been implicated in cancer development: NRF2-mediated upregulation of the iron storage protein ferritin or heme oxygenase 1 can lead to enhanced proliferation and therapy resistance. Of note, NRF2 activation and alterations to iron signaling in cancers may hinder efforts to induce the iron-dependent cell death process known as ferroptosis.

***Critical Issues:*** Despite growing recognition of NRF2 as a modulator of iron signaling, exactly how iron metabolism is altered due to NRF2 activation in normal physiology and in pathologic conditions remains imprecise; moreover, the roles of NRF2-mediated iron signaling changes in disease progression are only beginning to be uncovered.

***Future Directions:*** Further studies are necessary to connect NRF2 activation with physiological and pathological changes to iron signaling and oxidative stress.

## Introduction

Before the Great Oxygenation Event (GOE) that took place around 2.4 billion years ago, the earth was a low-oxygen stew of acidic oceans rich in dissolved ferrous iron (85). The onset of GOE was initiated when cyanobacteria and their evolutionary ancestors began utilizing photosynthesis to produce molecular oxygen (O_2_) by splitting water molecules. The increased O_2_ oxidized the ferrous iron and resulted in the rusting of the earth. Today, evidence of the oxidized iron deposits can be seen in geological deposits as banded red-colored iron minerals (20). Only following oxidation of the ferrous iron sinks could oxygen accumulate to the ∼21% atmospheric concentration seen in today's atmosphere. This profound interaction among iron, oxygen, and life persists to the present day where living organisms evolved systems to manage and exploit iron in redox chemistry.

Iron is an essential nutrient in the diet and is involved in a variety of critical intracellular processes, including DNA synthesis and cellular respiration. Iron serves as a cofactor in many enzymes involved in these processes due to its relative abundance on earth and its chemistry allowing easy participation in reduction and oxidation reactions; indeed, organisms from archaea to humans depend on iron-containing proteins (2). Despite the value of this reactivity in performing enzymatic catalysis and electron transfers, iron can also generate the highly reactive hydroxyl radical (OH^•^) *via* the Fenton reaction, which can damage lipids, proteins, and nucleic acids (29, 126, 145, 164):

\begin{align*}
F{e^{2 + }} + {H_2}{O_2} \to F{e^{3 + }} + O{H^ - } + O{H^ \cdot }
\end{align*}

Aside from iron, hydrogen peroxide feeds the Fenton reaction; H_2_O_2_ can form endogenously in biology by a variety of processes ranging from metabolic by-products to immune-related oxidases (159). Thus, cells maintain an appropriately sized pool of intracellular iron to balance its beneficial catalytic functions with deleterious free radicals by carefully regulating storage of iron, export of iron, degradation of major iron-containing proteins, and recycling of iron back into biologically valuable cofactors available to redox-active proteins.

**FIG. 1.**
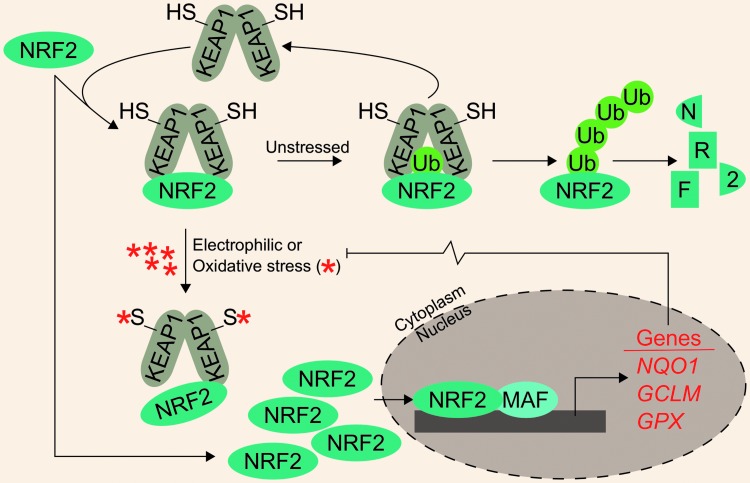
**KEAP1 regulates NRF2 degradation.** Under unstressed conditions, KEAP1 homodimers facilitate NRF2 ubiquitylation, which marks it for proteasomal degradation. Following NRF2 ubiquitylation, KEAP1 is recycled to bind newly synthesized NRF2. Under conditions of oxidative or electrophilic stress, key cysteine residues on KEAP1 are covalently modified, preventing it from mediating NRF2 ubiquitylation. Newly synthesized NRF2 can then accumulate and translocate to the nucleus where it dimerizes with one of the small MAF proteins to promote the transcription of cytoprotective genes. GCLM, glutamate-cysteine ligase modifier subunit; GPX, glutathione peroxidase; KEAP1, Kelch-like ECH-associated protein 1; MAF, musculoaponeurotic fibrosarcoma; NRF2, nuclear factor (erythroid-derived 2)-like 2.

Apart from iron-mediated oxidative stress, cells must also cope with a variety of electrophilic and oxidative xenobiotic stressors. These may include metabolites from heterotrophic consumption of other organisms or inorganic and organic environmental stressors. Development of antistress systems to ameliorate these toxic exposures was likely critical in early evolutionary development. One such system is the Kelch-like ECH-associated protein 1–nuclear factor (erythroid-derived 2)-like 2 system (KEAP1-NRF2), which is theorized to have arisen around the GOE (32). In this system, homodimers of KEAP1 negatively regulate the NRF2 transcription factor by targeting it for ubiquitylation and subsequent proteasomal degradation. As expected, NRF2 protein turnover is constant and quick under unstressed conditions. Following electrophilic and oxidative stress, cysteine residues on KEAP1 are modified; this induces a conformational change in KEAP1, disrupting its interaction with NRF2 and thus preventing NRF2 degradation. Newly synthesized NRF2 accumulates, translocates to the nucleus, dimerizes with small musculoaponeurotic fibrosarcoma (MAF) proteins, and facilitates transcription of a battery of Phase II metabolism and cytoprotective genes. For example, bona fide NRF2 target genes include NADPH quinone dehydrogenase 1 (*NQO1*), glutamate–cysteine ligase modifier subunit (*GCLM*), and the peroxide-scavenging selenoproteins of the glutathione peroxidase (GPX) family (Fig. 1).

Many reviews have focused on NRF2-mediated cytoprotection as well as KEAP1 and non-KEAP1-mediated NRF2 activation (34, 38, 56, 58, 86, 171). Mammalian NRF2 appears to have arisen from an ancestral NRF protein tracing back to eumetazoan lineages. Considering the ancient heritage of both NRF2 and iron regulation and their mutual crossover in the arena of intracellular stress, we sought to assess the literature to uncover relationships between NRF2 and iron.

## NRF2 Was Discovered for Its Roles in Erythropoiesis

Dedicated oxygen carriers are critical for the existence of multiple phyla in the metazoan kingdom. As its name suggests, the birth of research into nuclear factor (erythroid-derived 2)-like 2 (NFE2L2, NRF2) is rooted in erythropoiesis: the production of red blood cells or erythrocytes. The core function of erythrocytes is as an oxygen carrier that delivers oxygen to tissues for use in aerobic respiration. To do so, erythrocytes are rich in the iron metalloprotein, hemoglobin, which utilizes heme-bound iron to bind and transport oxygen. To better understand the production of hemoglobin, investigators were attempting to understand the transcriptional regulation of the hemoglobin subunits, α and β. The locus control region located 5′ of the β-globin gene cluster was known to harbor binding sites for ubiquitous and erythroid-specific transcription factors, including the promiscuous AP-1 binding site. One transcription factor, nuclear factor erythroid 2 (NF-E2), was shown to bind to the AP-1 site (99) and mediate globin gene expression (102, 110, 111, 148, 149). To fully characterize all DNA-binding proteins that could interact with the NF-E2 binding site, Moi *et al*. utilized a cDNA expression library from K652 cells to isolate several DNA-binding proteins that bound to the AP-1/NF-E2 repeat found in the locus control region of β-globin. One of the identified proteins that could bind to this region was NRF2 (101).

Although NRF2 was discovered as part of an effort to understand globin gene regulation, investigations into NRF2 and erythropoiesis quickly diminished for two reasons. First, within 2 years of its discovery, Chan *et al*. generated *Nrf2* knockout mice. Homozygous *Nrf2* knockout (*Nrf2^−/−^*) mice showed no visible phenotypes: mice exhibited no defects in embryogenesis, were fertile, and produced normal litter sizes; most importantly, hematological markers of *Nrf2^−/−^* mice did not differ from heterozygous or wild-type mice (15). Second, any roles of NRF2 in erythropoiesis were marginalized following the seminal works by Itoh *et al.* that demonstrated Nrf2 was essential for orchestrating the transcriptional induction of phase II detoxification genes carrying an antioxidant response element (ARE) (51). Indeed, Nrf2 was found to regulate ARE-carrying genes. Since then, much of the NRF2 scholarship has sought to characterize its roles in detoxification and electrophilic stress mediation.

Despite a shift from erythropoiesis to toxicology, studies suggest that NRF2 target genes may still be involved in erythropoiesis. Several NRF2 target genes have been directly implicated as anabolic enzymes necessary for hematopoietic cell maturation. In the original study characterizing mouse Nrf2 as well as subsequent analyses of the β-globin locus control region, *in vitro* reporter assays indicated that ectopically expressed Nrf2 can increase the expression of a reporter under the control of the globin enhancer region (93, 101), indicating that Nrf2 may participate in globin transcription. More recent studies have confirmed NRF2 binding to β-globin promoter regions using chromatin immunoprecipitation (ChIP) in lymphoblastoid cells treated with 10 μ*M* sulforaphane (SFN), an isothiocyanate found in cruciferous vegetables that is routinely used to activate NRF2. Notably, NRF2 may exhibit tissue-specific β-globin DNA binding and transcriptional induction (14). Others have extended the globin analyses to human fetal hemoglobin, which consists primarily of γ-globin, by showing that the NRF2 inducers, tert-butylhydroquinone (tBHQ, 25 μ*M*), D3T (25 μ*M*), and curcumin (10 μ*M*), could induce γ-globin mRNA. Moreover, tBHQ increased the protein level, nuclear localization, and γ-globin promoter binding of NRF2, which led to increased human fetal hemoglobin (88), indicating a role for NRF2 in globin production. While globin is necessary in many organisms for oxygen transport, the globin active sites require heme moieties to bind oxygen; thus, understanding how heme synthesis can be regulated by NRF2 provides a more complete understanding of relationships between iron, oxygen, and NRF2.

## NRF2 Regulates Genes Involved in Heme Biosynthesis

The profound interactions among iron, oxygen, and life are embodied in heme, a porphyrin-bound iron that is the center of many metabolic enzymes (mitochondria complexes and cytochrome P-450 enzymes), nitric oxide signaling effectors (nitric oxide synthases), oxygen storage proteins (myoglobin), and oxygen carrier proteins (hemoglobin). Of note, heme is required for oxygen transport over large distances because of poor dissolution of oxygen in aqueous solutions, including blood plasma. Thus, development of complexes capable of binding oxygen, such as heme, was critical for the evolution of large multicellular organisms; indeed, heme-bound iron accounts for ∼95% of iron within the human body (46). Heme is generated from the anabolic combinations of eight succinyl-CoA and eight glycine molecules over eight biosynthetic steps; in the final stages of heme synthesis, a single ferrous iron is inserted into a protoporphyrin to generate heme. The iron atom maintains four coordinate bonds to the rest of the heme prosthetic group, leaving two coordinate bonds available for protein interaction (*via* a histidine residue) and oxygen binding. The iron within heme can then bind oxygen for transportation, participation in redox reactions, or electron passaging. However, because iron can promote the formation of damaging oxygen radicals, synthesis and destruction of heme-bound iron are carefully regulated; NRF2 participates in the transcriptional regulation of heme metabolism.

Recent works have identified novel NRF2 target genes involved in heme biogeneration: ATP binding cassette subfamily B member 6 (ABCB6) and ferrochelatase (FECH). ABCB6 imports porphyrins, such as coproporphyrinogen III, from the cytosol to the mitochondria for further heme anabolism (70, 137). The *ABCB6* gene also encodes the Lan blood group antigen (42). NRF2 binding to *ABCB6* promoter regions was first identified in microarray analyses of airway epithelia from smokers (48). A putative NRF2-ABCB6 relationship was further characterized when knocking down *NRF2* in lung-derived cell lines, A549 and BEAS-2B, and liver-derived cell line, HepG2, treated with 10 μ*M* of sulforaphane showed ∼70% decreases in *ABCB6* transcript levels. Additionally, treatment with 10 μ*M* sulforaphane showed a fivefold induction of *ABCB6* mRNA in GM12878 lymphoblastoid cells. However, the same treatment did not induce *ABCB6* transcripts in bone marrow K562 cell lines, indicating that NRF2 modulation of ABCB6 may be tissue specific (14).

**FIG. 2.**
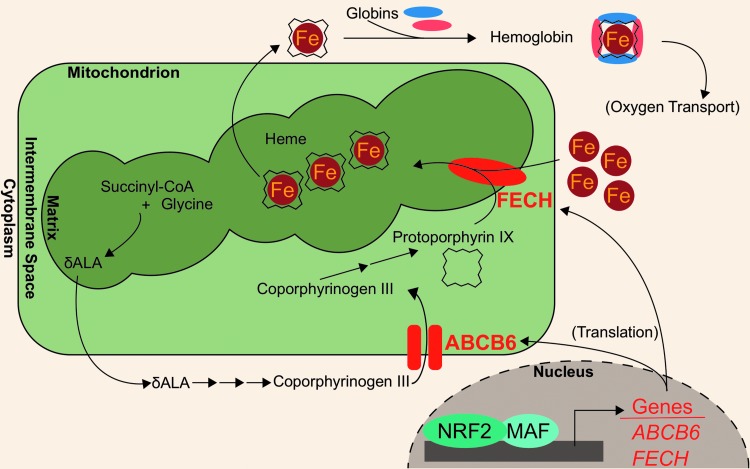
**NRF2 regulates heme synthesis.** Several genes involved in the heme biosynthetic pathway are transcription targets of NRF2 (*red-colored* genes). These genes include ABCB6, which transports coporphyrinogen III from the cytosol to the mitochondrial intermembrane space, and FECH, which inserts ferrous iron into the protoporphyrin ring. ABCB6, ATP binding cassette subfamily B member 6; FECH, ferrochelatase.

In addition to ABCB6, FECH is located in the mitochondria and participates in heme biosynthesis; in the last step of heme biosynthesis, ferrochelatase inserts ferrous iron into protoporphyrin IX to generate the final heme cofactor (60). Wu *et al*. identified ferrochelatase as an NRF2 target using a gene dose–response model. In this model, they compared gene expression profiles of *Nrf2*-null mice, *Nrf2*-wild-type mice, *Keap1*-knockdown mice, and hepatic *Keap1*-knockdown mice. After showing that these models have increasing liver Nrf2 activation, they identified a modest (<2-fold induction), but significant, induction of the *Fech* gene in the *Keap1*-knockdown and hepatic *Keap1*-knockdown mice relative to the *Nrf2*-null and *Nrf2*-wild-type mice (165). The role of NRF2 in mediating *FECH* gene expression was further evaluated in human cell lines, whereby *NRF2* knockdown in sulforaphane-treated (10 μ*M* sulforaphane for 24 h) A549, BEAS-2B, and HepG2 cells decreased *FECH* mRNA by 42%, 29%, and 62%, respectively (14). Consistently, treatment with 10 μ*M* sulforaphane increased *FECH* mRNA expression in GM12878 lymphoblastoid cells. However, NRF2-mediated FECH induction may be tissue specific, similar to ABCB6: neither sulforaphane treatment nor *KEAP1* knockdown in K562 bone marrow cells showed increased *FECH* expression (14). The NRF2-regulated aspects of heme anabolism are shown in Figure 2.

## NRF2 Regulates Genes Involved in Heme Catabolism

While Nrf2 regulates biological utilization of oxygen and iron by facilitating the transcription of genes that incorporate iron into heme during hemoglobin anabolism, NRF2 also regulates mobilization of iron from heme during hemoglobin catabolism. Senescent red blood cells are degraded by the mononuclear phagocyte system, consisting of primarily splenic macrophages and liver Kupffer cells. Following engulfment, erythrocytes are degraded in the phagolysosome and hemoglobin is degraded by proteases to liberate the heme cofactor (10). The heme is then exported out of the lysosome into the cytosol by the lysosomal membrane-bound transporter heme-responsive gene 1 (SLC48A1, HRG1) (127, 162). Knocking down *NRF2* in A549, BEAS-2B, and HepG2 cells decreases *HRG1* expression up to 52%, while activation of NRF2 by either *KEAP1* knockdown or treatment with 10 μ*M* sulforaphane results in increased *HRG1* mRNA expression by two- to sixfold and an increased HRG1 protein level (14). Although NRF2 activation appears to mediate HRG1 transcript and protein levels, it remains unclear to what extent heme trafficking and catabolism change.

Within macrophages, cytosolic heme from red blood cells is metabolized into ferrous iron and biliverdin by heme oxygenase (HMOX-1). Biliverdin is further metabolized, while the ferrous iron is recycled for fresh erythrocyte production. Before the discovery of Nrf2-mediated HMOX-1 regulation, *Hmox-1* mRNA was known to increase following administration of phorbol esters, sodium arsenite, H_2_O_2_, ultraviolet light, heme, and sulfhydryl compounds to skin fibroblasts. The RNA synthesis inhibitor actinomycin D has been shown to inhibit *Hmox-1* transcript induction following administration of many of these stimuli, thus implicating transcription as the key regulatory component for *Hmox-1* induction (17). Two putative distal enhancer regions at −4 and −10 kb were known to be required for induction by several stimuli, including heme, arsenite, and H_2_O_2_. They contained stress response elements (StREs), which were critical for inducer-dependent upregulation of *Hmox-1* transcription (3). As the StRE consensus sequence closely aligned with the binding site for the AP-1 groups of transcription factors, investigations focused on the well-studied Jun and Fos families of factors. However, the AP-1 site was identified to be only necessary, but not sufficient, for HMOX-1 induction by heme and cadmium; an extended AP-1 site with additional proximal three base pairs was deemed sufficient, which closely mirrored an ARE (49). Soon after, Alam *et al.* identified Nrf2 as the dominant mediator for *Hmox-1*. They showed that the expression of a dominant negative NRF2 mutant could inhibit *Hmox-1* mRNA induction by multiple agents (10 μ*M* heme, 100 μ*M* arsenite, 10 μ*M* cadmium, 100 μ*M* zinc, or 50 μ*M* tBHQ) by >85%, refuting the Jun/Fos model (4). These studies defined NRF2 as the main transcriptional regulator of HMOX-1. For a more robust discussion on the implications of NRF2 and HMOX-1 in health and disease, a comprehensive recent review has been conducted (83).

Further studies have demonstrated that a transcriptional repressor, BRCA-1-associated carboxy-terminal helicase (BACH1), can occupy the same binding sites on *HMOX-1* as NRF2. Bach1 is a BTB-basic leucine zipper transcription factor that antagonizes small Maf proteins that activate transcription at NF-E2 sites, including Nrf2 (119). Notably, Bach1 is a heme sensor; upon binding heme, Bach1 is ubiquitylated and targeted for proteasomal degradation (170). It was shown that Bach1 must be displaced before Nrf2 binding to the *HMOX-1* gene (129), indicating that iron-containing hemin and Nrf2 cooperate to induce HMOX-1.

While free iron generated from heme catabolism can be utilized or stored by the cell, the remaining heme catabolite, biliverdin, is metabolized to bilirubin by either biliverdin reductase A (BLVRA) or biliverdin reductase B (BLVRB). Bilirubin subsequently serves as an antioxidant free radical scavenger and can be glucuronidated for excretion. Transcription of BLVRB increased following Nrf2 activation; mice with liver-specific *Keap1* knockout showed 270% increase of *Blvrb* mRNA (165). Human BLVRB was also shown to be an NRF2 target gene: both *KEAP1* knockdown and treatment with 15 μ*M* sulforaphane induced *BLVRB* transcripts one- to threefold in breast cancer MCF10A cells (1). ChIP-seq and microarray analyses were combined to demonstrate that 100 μ*M* diethyl maleate targeted Nrf2-MafG binding to a site proximal to the *Blrvb* gene locus, and the *Blrvb* gene transcripts approximately doubled (44). Similarly, 15 μ*M* sulforaphane enhanced transcript and protein levels of BLVRA by one- and fivefold, respectively (1). *Hmox-1* and both BLVRs appear to depend on NRF2, indicating that NRF2 plays a critical role in heme catabolism; indeed, NRF2 critically mediates cross talk between these two systems: BLVRA can bind heme and transport it into the nucleus to induce *Hmox-1*, likely by delivering heme to the Bach1 repressor and abrogating Bach1 activity (157). Like many of the heme or erythropoietic genes associated with NRF2 activity, definitive correlation of the NRF2 role in modulation of BLVRA or BLVRB transcription with a physiological or pathological outcome is wanting.

Aside from BLVRs, NRF2 has also been implicated in the transcription of another enzyme that protects cells against oxidation by heme: alpha-1-microglobulin (A1M). The gene for A1M is alpha-1-microglobulin/bikunin precursor (*AMBP*); following cleavage by a protease, furin, AMBP gives rise to both A1M and bikunin, an extracellular matrix component. While the precise reactions of A1M remain unknown, A1M has cytoprotective and antioxidant effects due to its reported multifaceted role as a reductase/dehydrogenase, heme-binding and degrading protein, and free radical scavenger (114). In erythroid cells, A1M prevents intracellular oxidation and can bind heme (96, 115, 141). The proposed antioxidant mechanisms also include upregulation of its own transcription in response to hemoglobin, heme, and reactive oxygen species (ROS). These functions have been recently reviewed elsewhere (114). SFN treatment (10 μ*M* for 8 h) of K562 chronic myeloid leukemia cells and GM12878 lymphoblastoid cells significantly increased *AMBP* expression by 2- and 56-fold, respectively. As expected, when *KEAP1* was knocked down in K562 cells, *AMBP* mRNA increased more than twofold. Knocking down *NRF2* in A549 lung adenocarcinoma cells and in sulforaphane-treated (10 μ*M*) BEAS-2B bronchial epithelial cells and HepG2 hepatocellular carcinoma cells showed decreased *AMBP* transcript levels (14). Thus, NRF2 may mediate protection against heme- and hemoglobin-induced reactive oxygen species by promoting *AMBP* expression.

**FIG. 3.**
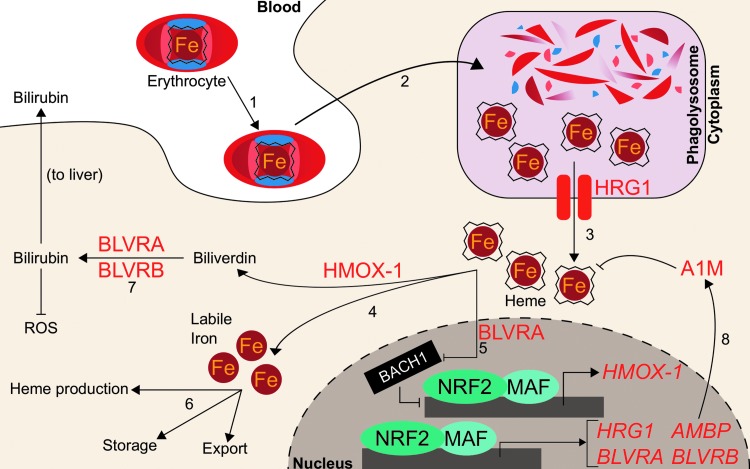
**NRF2 regulates heme degradation.** Putative NRF2 transcription targets are in *red-colored text*. (1) Aged erythrocytes, which contain hemoglobin, are endocytosed by macrophages. (2) Endocytosed erythrocytes are degraded in the phagolysosome of macrophages to liberate heme. (3) Heme is exported from the lysosome to the cytosol by HRG1, a transcriptional target of NRF2. (4) HMOX-1, a transcriptional target of NRF2, catabolizes heme into biliverdin and free iron. (5) BLVRA, a transcription target of NRF2, represses BACH1 and allows NRF2 to promote HMOX-1 expression. (6) Free iron liberated from heme is recycled for further heme synthesis, storage, or export from the cell. (7) Biliverdin is metabolized to bilirubin by BLVRA and BLVRB, which are transcription targets of NRF2. Bilirubin is then excreted to blood circulation and transported to the liver, where it is excreted as bile. (8) The NRF2 transcription target AMBP is proteolytically cleaved to A1M, which can bind heme. A1M, alpha-1-microglobulin; AMBP, alpha-1-microglobulin/bikunin precursor; BACH1, BRCA-1-associated carboxy-terminal helicase; BLVRA, biliverdin reductase A; BLVRB, biliverdin reductase B; HMOX-1, heme oxygenase; HRG1, heme-responsive gene 1; ROS, reactive oxygen species.

While NRF2 may participate in the safe destruction of heme *via* its induction of *HRG1*, *HMOX-1*, and the BLVRs, it may also participate in the incorporation of iron back into heme *via* its upregulation of genes such as *FECH* and *ABCB6*. These complementary roles could place NRF2 as a regulator at both the birth and death of iron recycling for erythrocyte production. Because iron participates in Fenton reactions, cells may utilize the redox-sensitive KEAP1-NRF2 system to ensure safe handling of the massive quantities of iron used during the generation and destruction of heme necessary for oxygen-facilitated biology. More dissection of the role of NRF2 in altering heme metabolism, quantity, or function is required in future studies. The NRF2-regulated heme homeostasis is summarized in Figure 3.

## Other Roles of NRF2 in Erythropoiesis

Although NRF2 appears to regulate many genes involved in hemoglobin metabolism and iron utilization, *Nrf2^−/−^* mice did not show anemia (15). However, follow-up studies indicated that older *Nrf2^−/−^* mice had signs of anemia and presented with splenomegaly and spleen toxicity. Indeed, hematological analyses showed that *Nrf2^−/−^* mice had abnormal erythrocyte morphology and were more sensitive to H_2_O_2_-induced hemolysis (79). Whether these defects are due to decreased functional hemoglobin, decreased detoxification, or some combination thereof remains unclear. It is possible that alterations to red blood cell function in *Nrf2^−/−^* mice may arise from decreased antioxidant capacity in erythrocytes.

Substantial evidence indicates that accumulation of ROS is particularly deleterious to red blood cells and can lead to hemolysis (30, 33, 52). As expected, ablation of ROS-scavenging selenoproteins, such as the GPXs, can induce anemia. Red blood cell count, hemoglobin concentration, and hematocrit in mice with Cre-Lox-induced deletion of the tRNA^sec^ gene *Trsp*, which normally allows synthesis of selenoproteins, were decreased to ∼60% of the control. Moreover, combined inactivation of GPX selenoproteins and Nrf2 exacerbated the anemia: the same hematopoietic parameters dropped to 30% of the control. The combined loss of selenoproteins and Nrf2 resulted in increased erythrocyte intracellular ROS levels. Thus, the battery of Nrf2 selenoprotein genes critically regulates redox homeostasis in erythrocytes and may prevent hemolytic anemia (57).

The antioxidant functions of GPX proteins are complemented by a host of other antioxidant defense systems that can be activated by NRF2. Broadly, these include the peroxiredoxin system (18, 64), the thioredoxin system, and the glutathione system. NRF2 has been shown to facilitate the transcription of enzymes involved in these defense systems, as reviewed recently (39, 173). Importantly, players in these systems such as the peroxiredoxins have been shown to be particularly efficient ROS scavengers (9, 55) capable of affecting both physiological iron homeostasis and erythrocyte health (95, 109). Excellent reviews encompassing the NRF2-peroxiredoxin system are available (35, 39, 150, 174).

Beyond its role in regulating redox stress during hematopoietic cell development, studies have also shown that NRF2 plays a role in hematopoietic stem cell (HPSC) maintenance (105). The HPSCs in *Nrf2^−/−^* mice exhibit a 65% increase in apoptosis compared with *Nrf2^+/+^* mice, which was not associated with an increase in ROS; moreover, Nrf2 appears to balance HPSC proliferation, self-renewal, and bone marrow localization (97, 155). Nrf2-mediated Notch signaling improved HPSC function following ionizing radiation (62). Murakami *et al*. demonstrated the role of Nrf2 in mediating HPSC fate by using *Keap1*-deficient mice, which presented with almost 10% enhanced granulocyte–monocyte differentiation and compensatory decrease in erythroid and lymphoid differentiation. *Keap1^−/−^* HPSCs mimicked the nonerythroid lineage priming, while combined *Nrf2/Keap1* knockout restored lineage commitment (106). Given that genes important for heme biosynthesis, such as *ABCB6* and *FECH*, are transcription targets of NRF2, it seems surprising that NRF2 activation favors nonerythroid lineages that depend less on heme. To further complicate the role of NRF2 in erythropoiesis, some roles of NRF2 in HPSC maintenance are proposed to be independent of oxidative stress (97), while other studies indicated that NRF2 protects erythrocytes from oxidant-induced hemolysis and anemia (79). Clearly, erythropoiesis is a carefully regulated process with many players and processes, and NRF2 mediates erythropoietic processes, including cellular antioxidation, stem cell maintenance and differentiation, and transcriptional activation of heme anabolic genes. The apparently conflicting roles of NRF2 within many hematological processes require further dissection.

## NRF2 Compartmentalizes Nonheme-Associated Iron

Apart from heme-bound iron, cells maintain a pool of labile (redox-active, exchangeable, and chelatable) iron to be used for biosynthetic processes such as heme or iron–sulfur cluster generation. Labile iron is very tightly regulated to a cytosolic concentration of 0.5–1.5 μ*M* at homeostasis, which comprises <5% of total intracellular iron (12): as mentioned previously, labile iron can enable the formation of oxygen-derived free radicals, such as the highly damaging hydroxyl radical, through Fenton reaction.

The product of Fenton reaction, OH^•^, is a potent oxidizer that rapidly reacts with nearby proteins and lipids. Its high reactivity gives OH^•^ a short half-life in biological systems on the order of 1 ns (67). Physiological damage from iron-associated (Fenton reaction-derived) hydroxyl radicals is extensively limited at the physiological level because of the relatively low homeostatic levels of labile iron, which are maintained by a cellular buffering system.

A central player in the iron-buffering system is the ferritin protein, an ancient intracellular iron storage protein common to all five kingdoms of life (6). Ferritin sequesters excess free iron in a protein cage that limits iron's redox switching. Human ferritin consists of 24 subunits comprising ferritin heavy chain (FTH1) and ferritin light chain (FTL) polypeptides (68). The ratio of FTH1 to FTL can vary widely by tissue or disease state (8), and the functions of each subunit also differ: FTH1 contains a ferroxidase active site, which can oxidize Fe^2+^ to Fe^3+^ for storage in the central core, while FTL is the primary stabilizer of the ferritin protein and thus can dominate the FTL/FTH1 ratio in tissues such as the liver, where long-term iron storage is necessary (152). Ferritin readily sequesters up to 4500 iron atoms into its core (87), preventing the iron from participating in Fenton reactions. Thus, increased ferritin expression can have an antioxidant effect. In times of iron deficiency, the iron reservoir within ferritin proteins can be liberated through ferritinophagy (90, 91). At the same time, translation of ferritin mRNAs is repressed by iron regulatory proteins (IRPs), which bind to iron response elements (IREs) located in the 5′ untranslated regions (UTRs). IRPs comprise IRP1 and IRP2 and their intracellular levels are responsive to the labile iron pool. High labile iron pool levels lead to destruction of IRPs through proteasomal degradation, while low labile iron stabilizes them (103, 104, 132, 158, 163). Ferritin thus serves as a reservoir to maintain an appropriately sized labile iron pool for biosynthetic processes.

While NRF2 assists with maintaining physiological iron homeostasis due to its roles in regulating heme-bound iron, NRF2 also plays a pivotal role in regulating iron homeostasis within the labile iron pools. The earliest and most striking phenotypic examples of NRF2 effects on iron storage and transportation followed the discovery that *Nrf2*^−/−^ mice showed abnormally white teeth compared with *Nrf2^+/+^* mice; this color switch was determined to be due to defective iron utilization in developing tooth enamel of *Nrf2^−/−^* mice (167). Many investigations have subsequently identified and characterized specific genes involved in management of nonheme iron. One of its most important functions in this regard is as a transcriptional activator of ferritin.

The first indication of Nrf2 as a transcription factor that modulates *Fth1* and *Ftl* transcription arose when depletion of glutathione in rat livers was shown to increase both *Ftl* and *Fth1* transcripts (13). Shortly after, AREs were identified within the promoter regions of murine *Ftl* and *Fth1* genes (156, 161). However, it was unknown which transcription factors could bind to the AREs or if that binding affected transcription. The heavy chain of ferritin, *Fth1*, was first identified as an Nrf2 target gene when basal *Fth1* mRNA levels in *Nrf2*-deficient mice were lower than in *Nrf2*-wild-type mice (74). The relationship between Nrf2 activation and *Ftl* was soon uncovered when *Ftl* transcripts were twofold higher in *Nrf2^+/+^* compared with *Nrf2*-deficient mouse intestines (153). These two studies provided the first link between Nrf2 and *Ftl* and *Fth1* transcription; however, neither study found that Nrf2-activating xenobiotics could induce *Ftl* or *Fth1* and instead proposed that Nrf2 functioned solely to enhance basal transcription of ferritin. While chemopreventive xenobiotics such as the dithiolethiones oltipraz and 1,2-dithiole-3-thione had been known to activate Nrf2 (73) and induce ferritin (125), nobody had directly linked xenobiotic activation of Nrf2 to ferritin induction. Pietsch *et al*. connected these concepts by showing that both *Ftl* and *Fth1* could be induced approximately twofold by 70 μ*M* of various dithiolethiones only in the presence of Nrf2 in primary mouse embryo fibroblasts (123). They demonstrated using gel shift assays that Nrf2 directly bound to the known *Fth1* ARE following administration of chemopreventive dithiolethiones oltipraz and 1,2-dithiole-3-thione (123). Thus, Nrf2 is responsible for basal transcription of ferritin, and xenobiotic-activated Nrf2 can induce ferritin beyond its normal levels.

While upregulation of the ferritin gene transcription by NRF2 alters iron homeostasis by increasing iron storage and decreasing labile iron, NRF2 also buffers labile iron by altering its flux in and out of the cell. NRF2 does so by modulating the expression of ferroportin (FPN1), the only known mammalian exporter of iron from the cytosol to the extracellular milieu. FPN1 exports iron from iron-absorbing enterocytes of the intestinal lining into the bloodstream, thus adjusting the amount of dietary iron entering circulation (27). Similarly, FPN1 is responsible for iron mobilization from the hemoglobin-recycling macrophages and iron-storing hepatocytes (27). As iron is only normally lost due to bleeding, menstruation, childbearing, or intestinal cell sloughing, the aforementioned processes are required to ensure adequate iron supply for cellular functions and hemoglobinization without inducing iron overload and the associated oxidative stress. The role of Nrf2 in Fpn1 regulation arose from studies on macrophages in mice. Multiple groups showed that macrophage mRNA levels of *Fpn1* increased following erythrophagocytosis (22, 65). No transcription factors were identified facilitating the response to erythrophagocytosis until Marro *et al*. identified that the transcriptional repressor Bach1 controls *Fpn1* expression. Tenfold induction of *Fpn1* by heme was due to release of the heme-sensitive Bach1 repressor; subsequently, a putative ARE was identified approximately −7 kb from the *FPN1* core promoter. Accordingly, treatment with sulforaphane increased *Fpn1* mRNA expression more than fivefold (94). The current model proposes that Bach1 binds to the ARE until heme exposure, wherein Bach1 leaves the DNA and allows Nrf2 to bind in its place and promote *Fpn1* transcription. Notably, Nrf2 activation may simply displace Bach1 without Bach1 degradation. Regardless, heme inactivates Bach1 (113), thus providing a consistent regulatory element to Nrf2 activation of both HMOX-1 and FPN1, two proteins involved in heme catabolism and iron recycling. Additional evidence points to *Ftl* and *Fth1* regulation by Nrf2 requiring Bach1 inactivation (43).

Further studies demonstrated that several Nrf2 activators, including diethyl malate (100 μ*M*) and sulforaphane (5 μ*M*), can upregulate *Fpn1* mRNA in murine macrophages in an NRF2-dependent iron-independent manner. The increased Fpn1 was estimated to release significantly more (5% extra) intracellular iron to the extracellular milieu. Nrf2-mediated increases in iron export from macrophages were efficient enough to counteract lipopolysaccharide-induced suppression of *Fpn1* mRNA expression. This indicates that Nrf2 may regulate iron trafficking during inflammation *via Fpn1* expression (37). Thus, NRF2 may reduce intracellular labile iron pools by inducing expression of the iron storage ferritin proteins, FTL and FTH1, and the iron exporter FPN1. Further studies using an *in vivo* model are necessary to determine whether alterations to NRF2-mediated ferroportin induction can alter inflammation or infection outcomes.

While excess iron can be sequestered in ferritin or exported by ferroportin, iron can also directly impact cellular signaling following its incorporation into a protein known as pirin (PIR). PIR directly interacts with B cell lymphoma 3-encoded (Bcl-3) to coregulate nuclear factor kappa-light-chain-enhancer of activated B cell (NF-κB) transcriptional signaling. Crystal structures of PIR show it uses iron as a cofactor, suggesting PIR has an enzymatic redox function (120). PIR conformations change depending on whether the bound iron is Fe^2+^ or Fe^3+^, and the redox state of PIR iron alters its allosteric regulation of NF-κB DNA binding capabilities. It has been proposed that the NF-κB role in responding to intracellular oxidative stress may depend on the PIR redox state (80). PIR was initially identified as an NRF2 target following an investigation into differential gene expression between small airway epithelia of smokers and nonsmokers. Mobility shift assays showed direct NRF2 binding to putative AREs (48). NRF2-mediated *PIR* transcription was validated in HeLa cervical cancer cells: a functional ARE was identified at +281 bp downstream of the transcription start site. Additionally, *NRF2* knockdown in HeLa cells decreased PIR mRNA and protein levels. NRF2 overexpression increased *PIR* mRNA level 30% relative to that of control, and ChIP demonstrated NRF2 binding to *PIR* promoter ARE (11). Although the role of NRF2 in modulating PIR levels to alter physiology or disease progression is unclear, PIR and iron may serve as a pathway linking NRF2 and NF-κB transcriptional programs.

**FIG. 4.**
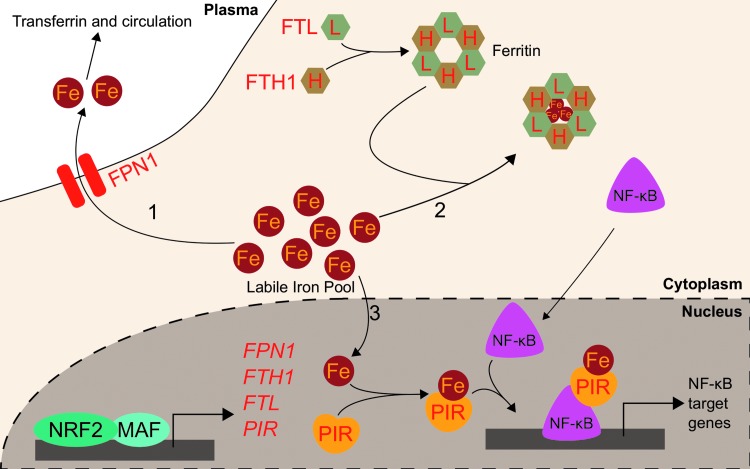
**NRF2 regulates the labile iron pool.** Putative NRF2 transcription targets are in *red-colored font*. **(1)** Labile iron can be exported out of the cytosol by FPN1, which is an NRF2 transcription target. **(2)** Labile iron can be sequestered in ferritin, which comprises FTL and FTH1 polypeptide subunits. Both *FTL* and *FTH1* are known transcription targets of NRF2. **(3)** Labile iron can bind to PIR and influence NF-κB transcriptional activity. FTH1, ferritin heavy chain; FTL, ferritin light chain; FPN1, ferroportin; NF-κB, nuclear factor kappa-light-chain-enhancer of activated B cell; PIR, pirin.

NRF2 activation is generally expected to reduce cytosolic labile iron: ferritin can sequester iron, ferroportin can export iron, and pirin can utilize iron as a cofactor for intracellular signaling. NRF2 activation can thus enable a reduction in the intracellular iron pool, restoring homeostasis in situations of cellular iron overload and preventing oxidative stress. NRF2-mediated regulation of labile iron pool is summarized in Figure 4.

## Iron Homeostasis, NRF2, and Cancer

Given that iron is an essential nutrient and iron-containing proteins play diverse roles in cell cycle, DNA repair, and metabolism, many proteins involved in modulating iron homeostasis are now implicated in cancer, including the aforementioned ferroportin and ferritin proteins (92, 154). For example, glioblastoma cells were found to be particularly dependent on high ferritin levels; knocking down ferritin *in vitro* reduced tumor cell proliferation by sevenfold. Xenograft studies showed that shRNA-mediated *FTH1* or *FTL* knockdown suppressed tumorigenesis of glioblastoma cells (136). Understanding how iron signaling becomes deregulated in cancer could lead to novel understanding of the tumor as well as fresh treatment avenues.

One approach is to understand how NRF2 is deregulated in cancer and how that in turn can alter iron homeostasis. Similar to many iron-modulatory proteins, NRF2 activation has also been implicated in cancer: NRF2 activation in some tumor types such as nonsmall cell lung cancer is associated with poor disease prognosis, including worse overall survival with hazard ratios of 1.75 for patients with NRF2 expression and 2.09 for patients with low or undetectable KEAP1 expression (98, 143). Current models posit that while transient NRF2 activation protects cells from deleterious insults and carcinogens, constitutive activation of NRF2 actually promotes carcinogenesis. Recent studies have highlighted this dark side to NRF2 activation in cancer (77, 124). For example, somatic mutations that activate NRF2 or inhibit KEAP1-mediated degradation of NRF2 have shown prevalence in many cancers (40, 138). Additionally, some environmental carcinogens such as arsenic are capable of constitutively activating NRF2 in nanomolar–micromolar exposure ranges (78, 122). Once activated, NRF2 can promote carcinogenesis by facilitating several classical hallmarks of cancer, including angiogenesis, metabolic reprogramming, chronic proliferation, and resistance to cell death (36, 41, 56). Additionally, one of the main consequences of constitutive NRF2 activation in tumors is its ability to upregulate cytoprotective genes that rapidly metabolize and eliminate chemotherapeutics and reduce treatment efficacy (160). While few investigations have unequivocally established relationships between dysregulated NRF2, iron, and oxygen in cancer, studies are beginning to uncover that NRF2 activation in cancer may promote the disease through its modulation of iron signaling.

Currently, the NRF2 iron-related target gene most strongly associated with cancer is *HMOX-1* (107). Several studies have indicated that inhibition or elimination of HMOX-1 in NRF2-activated cells sensitizes them to chemotherapeutics (107). For example, A549 cells, a lung cancer cell line with constitutive NRF2 activation, shows HMOX-1 overexpression relative to other lung cancer cell lines H23, H127, and H460. *NRF2* knockdown by RNA interference demonstrated that the high levels of HMOX-1 in A549 cells were due to its constitutive NRF2 (61). Importantly, high HMOX-1 expression decreases susceptibility to cisplatin chemotherapy; genetic or pharmacological inhibition of HMOX-1 boosts cisplatin cytotoxicity in A549 cells by 20%–50%. Mechanistically, HMOX-1 was proposed to decrease reactive oxygen species in the cells (61, 71). A similar effect was seen with a different proapoptotic compound, epigallocatechin 3-gallate (EGCG), in A549 cells. EGCG was found to upregulate HMOX-1, and following siRNA-mediated knockdown of *HMOX-1*, cells were significantly more susceptible to apoptosis when exposed to 50–100 μ*M* EGCG. Intriguingly, the study also found that 100 μ*M* of the iron chelator deferoxamine could reduce the EGCG-induced upregulation of HMOX-1, indicating a role for iron in resistance to chemotherapeutics (75). Several studies have expanded the carcinogenic roles of NRF2 and HMOX-1 beyond lung cancer. For example, NRF2-mediated HMOX-1 induction attenuated arsenic trioxide-induced cell death and ROS in glioma cells (82). NCI-H292 lung cancer cells overexpressing NRF2 and HMOX-1 showed an induction of thymidine phosphorylase and a subsequent increase in angiogenic potential as indicated by a 150%–200% increase in endothelial cell branching (151). Biallelic fumarate hydratase (*FH*) inactivation, which is the initiating event of hereditary leiomyomatosis and renal cell cancer (HLRCC), also activates NRF2 (116). In these cells, *HMOX-1* knockdown was also shown to be synthetic lethal with *FH* inactivation (31). Clearly, more information on exactly how NRF2-HMOX-1 contributes to cancer is needed.

Aside from HMOX-1, oncogenic relationships between ferritin and NRF2 are emerging. Much like HMOX-1, several studies have indicated that ferritin upregulation protects cells against chemotherapeutics such as doxorubicin or carmustine. For example, siRNA-mediated *FTH1* knockdown decreased the LD_50_ of carmustine from 100 to 40 μ*M* in U-251 glioblastoma cells, and similar depletion of FTH1 and FTL by miR-200b lowered the IC_50_ of doxorubicin from 20 to <5 μ*M* in MDA-MB-231 breast cancer cells (81, 140). Ferritin upregulation has been shown to increase resistance to tumor necrosis factor-induced cell death as well (121). Given that ferritin transcription is under the control of transcription factors beyond NRF2, such as NF-κB (72), the extent to which NRF2 specifically contributes to ferritin overexpression and associated chemotherapy resistance remains undetermined.

The roles of ferritin in cancer go beyond its role in intracellular redox stress and cell death. Recent studies on ferritin in cancer show that it can alter intracellular signaling. Schonberg *et al*. showed that high ferritin in glioblastoma cells induced a chronic proliferative signal by activating the promitotic Forkhead Box M1 transcription factor (FOXM1) (136). Our own group has pioneered the translation of this work into the NRF2 field through study of a hereditary cancer syndrome known as HLRCC (59). In HLRCC renal tumors, the oncometabolite fumarate constitutively activates NRF2 and inhibits IRPs (117). Thus, fumarate increases ferritin transcription and translation, leading to ferritin protein accumulation. HLRCC tumor cells subsequently rely on ferritin for FOXM1-mediated proliferation: ferritin knockdown ablated FOXM1 and abrogated cell growth (59). Whether NRF2 and FOXM1 signaling are connected through ferritin in cancers with genetic- or toxicant-induced NRF2 activation requires inquiry.

While the exact mechanism explaining how ferritin upregulation induces FOXM1 signaling is not well characterized, it could be through an iron chelation effect induced by iron-independent upregulation of ferritin. Should NRF2 activation induce intracellular iron deficiency, this could have profound implications for many biological iron-dependent carcinogenic processes, ranging from replication, DNA repair, metabolism, and iron-dependent dioxygenase activity (7, 21, 45, 84, 108, 112, 131, 134). Iron-dependent dioxygenases include the prolyl hydroxylases that negatively regulate the proangiogenic transcription factors: hypoxia-inducible factors 1α and 2α (HIF1α and HIF2α) (53). Inhibition of NRF2 has been shown to reduce HIF1α (54, 63), mirroring the activation of HIF1α seen with iron chelation treatment (53). Interestingly, upregulation of ferritin has been shown to lower available iron by an estimated 25%, decrease prolyl hydroxylase activity by ∼75%, and activate HIF1α (142). Thus, intracellular iron signaling can serve as a link between HIF and NRF2. Intriguingly, translation of *HIF2*α is also regulated by IRPs: akin to ferritin, *HIF2*α contains an IRE in its 5′ UTR that is particularly susceptible to translational inhibition by IRPs (5, 135). If the NRF2-mediated intracellular iron deficiency holds true, NRF2 activation will selectively activate HIF1α and not HIF2α, as the latter would have been repressed at the translation level. The differential activation of HIF1α *versus* HIF2α may have very different consequences. As was observed in renal cell carcinoma, HIF2α, but not HIF1α, confers tumorigenic potential upon inactivation of the von Hippel-Lindau tumor suppressor (128).

**FIG. 5.**
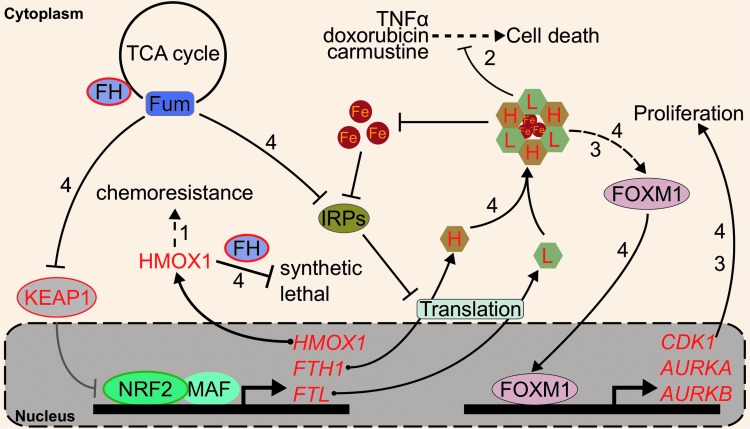
**NRF2, iron, and cancer.** NRF2 gain-of-function mutation (*green-bordered ellipse*) and KEAP1 loss-of-function mutation (*red-bordered ellipse*) are frequently found in cancer. These mutations lead to sustained NRF2 activation. **(1)** NRF2 promotes the expression of HMOX-1, which was previously shown to confer chemoresistance. **(2)** NRF2 promotes ferritin expression, which was shown to protect cells against TNFα-, doxorubicin-, and carmustine-induced cell death. **(3)** Increased ferritin level in glioblastoma cells was shown to promote FOXM1 activity and confer a proliferative phenotype. **(4)** In *FH* loss-of-function (*red-bordered ellipse*) HLRCC cancer cells, HMOX-1 knockdown was shown to be synthetic lethal with *FH* inactivation. In these cells, fumarate [Fum] inhibits KEAP1 and drives NRF2 activation. Additionally, fumarate also inhibits IRPs, which normally repress the translation of *FTL* and *FTH1*. This leads to intracellular ferritin accumulation that drives cellular proliferation through FOXM1 activation. NRF2 transcription targets are in *red*. *FH*, fumarate hydratase; FOXM1, Forkhead Box M1 transcription factor; HLRCC, hereditary leiomyomatosis and renal cell cancer; IRP, iron regulatory protein; TNF, tumor necrosis factor.

The interplay between transcriptional control of ferritin genes by NRF2 and translational control by IRP2 also merits exploration. KEAP1 regulation of NRF2 is mediated by a series of reactive thiols (23); different cysteines appear to react with different inducers to activate NRF2 (56, 66, 172). IRP2 also seems to show compound-dependent increases (59) or decreases (19, 175) to IRE binding, likely based on IRP2 cysteine modifications (176). Understanding the combined iron-relevant cysteine code that determines if and when ferritin protein increases could provide insight into the relationship between NRF2 and iron signaling. The roles of NRF2-mediated iron homeostasis regulation in cancer are summarized in Figure 5.

## NRF2 Regulates Sensitivity to Ferroptosis, an Iron-Dependent Cell Death Mechanism

The profound interactions between oxygen and iron create a check and balance system that keeps iron-associated oxidative stress in check while allowing cells to utilize the redox properties of iron to enable living processes. When thrown out of balance, iron-induced oxidative stress may induce sufficient cellular damage to prevent biological activity and cause cell death. Indeed, free iron and its associated reactive oxygen species are important initiators and mediators of cell death, as reviewed recently (25). One of the main biomolecule targets of iron-mediated hydroxyl radicals is lipid; following abstraction of lipid hydrogen by hydroxyl radicals, the newly formed lipid radical reacts with another nearby lipid to generate a lipid peroxide and a second lipid radical. This process propagates forward, generating a series of lipid peroxides that alter membrane fluidity and membrane-bound protein mobility.

NRF2, iron, and oxygen again cross paths upon interrogation of the NRF2 role in ameliorating lipid peroxidation. Activated NRF2 protects cells against hydroperoxides by upregulating the transcription of the GPX family of proteins. GPXs utilize monomeric reduced glutathione (GSH) to eliminate peroxides, generating water and oxidized glutathione as products. Of the GPXs, glutathione peroxidase 4 (GPX4) serves as the primary neutralizer of lipid peroxides (89).

Aberrant accumulation of lipid peroxides through inhibition of GPX4 or depletion of GSH has been shown to induce ferroptosis, an iron-dependent and nonapoptotic form of cell death (168). Ferroptosis was first discovered as the mechanism by which RAS synthetic lethal compounds, erastin and RSL3, selectively killed RAS-activated cells (24). Beyond its potential exploitation in cancer chemotherapy, ferroptosis is thought to contribute to tissue ischemic reperfusion injury, acute renal failure, and neurodegeneration. Iron itself is capable of inducing lipid peroxidation, both enzymatically and nonenzymatically, although the exact role of iron in ferroptosis is unclear (26, 145). Regardless, addition of iron chelators such as deferoxamine (DFO) or knockdown of the negative regulator of ferritin translation, IRP2, inhibits ferroptosis, indicating that ferroptosis is an iron-dependent oxidative cell death (24). To date, many ferroptosis-inducing (FIN) compounds have been identified, which generally fall into two classes. Class 1 compounds (*e.g.*, erastin) prompt ferroptosis through inhibition of SLC7A11, which is a cysteine–glutamate antiporter responsible for the import of extracellular cysteine required for GSH synthesis. Following SLC7A11 inhibition, GSH synthesis stops, GPX4 cannot eliminate lipid peroxides, and ferroptotic cell death commences. On the other hand, Class 2 FIN compounds (*e.g.*, RSL3) directly inhibit GPX4 to induce lipid peroxide accumulation and ferroptosis (168).

NRF2 activation has long been linked to protection against cell death (58), but investigators have only just begun to dissect the role of NRF2 in ferroptotic cell death and its relationship with iron signaling. Both *GPX4* and *SLC7A11* are bona fide NRF2 target genes (50, 118, 133), as are the glutathione synthesis genes γ-glutamylcysteine synthetase (*GCS*) (153), GCLM, and glutamate–cysteine ligase catalytic subunit (*GCLC*) (73), so NRF2 activation was expected to protect cells against ferroptosis. Additionally, degradation of FTL and FTH1, both NRF2 target genes, was shown to enhance ferroptosis; the logical assumption follows that NRF2-mediated induction of ferritin could desensitize cells to ferroptosis (47). One of the earliest indications that NRF2 and ferroptosis were related arose from a study that showed epicatechin protects against intracerebral hemorrhage by activating NRF2. In this study, the authors also identified that epicatechin reduced gene expression of critical ferroptosis regulators such as *IRP2*, which is not a target gene of NRF2. It remains to be uncovered how the NRF2-mediated ferroptosis protection interacts with concurrent IRP2 downregulation and how those pathways converge with iron proteins such as FTL or FTH1 (16). Soon after, NRF2 activation *via* p62-mediated inhibition of KEAP1 was shown to protect hepatocellular carcinoma cells against ferroptosis (147). Specifically, NRF2-mediated induction of the iron-related target genes *HMOX-1* and *FTH1* protected against ferroptosis; consistently, knockdown of either *HMOX-1* or *FTH1* by RNA interference enhanced ferroptotic cell death in hepatocellular carcinoma cells (147). Other genes that NRF2 regulated may modify ferroptosis sensitivity: NRF2-mediated induction of metallothionein-1G (MT-1G) has been shown to protect against ferroptosis. The ferroptosis inducer sorafenib more potently reduced the Huh7 hepatocarcinoma tumor burden in mice cotreated with MT-1G shRNA. Additionally, MT-1G knockdown in hepatocellular carcinoma cells nearly doubled erastin- and sorafenib-induced lipid peroxidation (146). NRF2 activation also contributes to ferroptosis resistance of head and neck cancer cells; importantly, *in vivo* inhibition of the NRF2 pathway sensitized ferroptosis-resistant head and neck cancers to 50 mg/kg of artesunate, a ferroptosis inducer (130). Cancer-preventive compounds, or chemopreventives, may also protect against ferroptosis: 10 μ*M* of the chemopreventive baicalein was shown to protect against ferroptosis by preventing NRF2 degradation and increasing GPX4 protein levels approximately threefold (166). Intriguingly, FINs appear to alter NRF2 levels themselves: the class 1 ferroptosis inducer erastin appeared to decrease NRF2 protein levels in pancreatic cancer and head and neck cancer cells (166), yet increased NRF2 levels in hepatocellular carcinoma cells (147). This mechanistic discrepancy merits further investigation to identify how different FINs affect NRF2 in different tissues. As many ferroptosis inducers concurrently activate NRF2, combining a ferroptosis inducer with an NRF2 inhibitor could provide a synergistic treatment option, although effective pharmacological NRF2 inhibitors remain elusive.

While most results thus far indicate that NRF2 plays a protective role against ferroptosis, some results indicate that pathways positively regulated by NRF2 can enhance ferroptosis. The ferroptosis inducer erastin was shown to induce expression of the NRF2 target gene HMOX-1 in HT-1080 sarcoma cells; inhibition of HMOX-1 with 10 μ*M* zinc protoporphyrin eliminated sensitivity to erastin-induced ferroptosis (76). Given that HMOX-1 has shown both pro- and antiferroptotic activities, more evidence is needed to discern if and when NRF2-mediated upregulation of HMOX-1 promotes or inhibits ferroptosis. Importantly, changes to intracellular iron levels and localizations must be appropriately investigated in the context of NRF2 activation and ferroptosis. Furthermore, NRF2 may regulate ferroptosis in a tissue-dependent manner; many of the iron-relevant NRF2 target genes described here show cell- or tissue-specific induction. To this end, we must understand the mechanistic transcriptional grammar underlying which target genes are activated by NRF2 under different conditions and tissues.

**FIG. 6.**
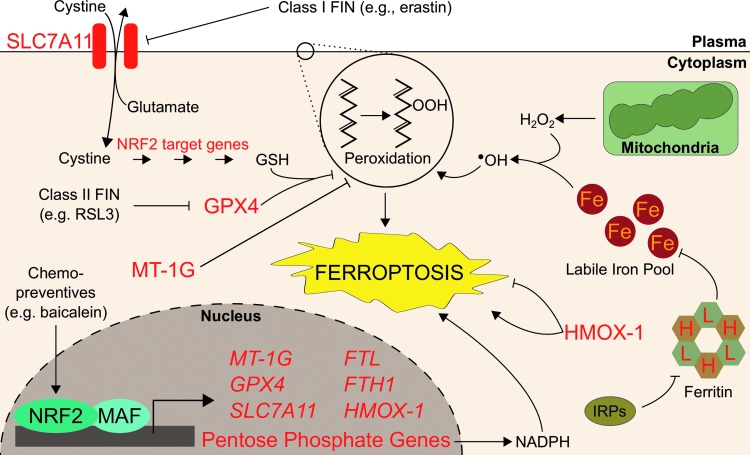
**NRF2 protects cells from ferroptosis.** Ferroptosis is an iron-dependent cell death inducible by FIN compounds. These compounds induce ferroptosis by directly or indirectly inhibiting GPX4 activities, causing cellular lipid peroxide accumulation and subsequent oxidative cell death. NRF2 protects cells from ferroptosis by increasing cellular glutathione production. NRF2 promotes glutathione synthesis by increasing expression of genes directly involved in the glutathione biosynthetic pathway. NRF2 also promotes the expression of SLC7A11, which increases intracellular cysteine pools, resulting in increased intracellular glutathione level. GPX4, which is the primary regulator of ferroptosis, is also an NRF2 target gene. NRF2 also regulates cellular iron availability by promoting the expression of ferritin (FTL and FTH1), which reduces the labile iron pool and thus protects cells against ferroptosis. Other NRF2 target genes, such as MT-1G and HMOX-1, have been shown to regulate ferroptosis. FIN, ferroptosis-inducing; GPX4, glutathione peroxidase 4; GSH, monomeric, reduced glutathione; MT-1G, metallothionein-1G; SLC7A11, solute carrier family 7 member 11/System xCT.

It also remains unclear how NRF2 interacts with other regulators of ferroptosis. While some negative regulators of ferroptosis are independent of NRF2, such as IRP2 or the DNA damage repair protein Fanconi anemia complementation group D2 (FANCD2) (144), other yet to be characterized players in ferroptosis may be partially regulated by NRF2. For example, NRF2 can upregulate expression of genes in the pentose phosphate pathway, pyruvate cycling (malic enzyme), and folate metabolism (*MTHFD2*) (100, 165), which can generate cellular NADPH (28, 153). While NADPH abundance has been determined as a biomarker for ferroptosis sensitivity (139), no investigations to date have determined the role of NRF2 in enhancing or ameliorating ferroptosis by mediating NADPH levels. Figure 6 summarizes the regulation of ferroptosis by NRF2.

## Conclusions

Since the GOE, iron and oxygen have been inexorably linked in biology. Ties between oxygen and iron are most exemplified in metabolism: aerobic organisms utilize oxygen for energy production while exploiting the redox properties of iron for oxygen transport, storage and tissue oxygenation (hemoglobin and myoglobin), oxidative phosphorylation electron shuttling (mitochondria proteins), and metabolite oxidation (cytochrome P450s). Despite the requirement for iron-mediated biology in beneficial oxidative processes, iron and oxygen can also damage cells: through the Fenton reaction, iron facilitates the production of oxygen-derived free radicals capable of damaging biomolecules. To mitigate the damaging aspects of iron and oxygen interactions, vertebrates place the bulk of their iron in the protein cofactor heme to appropriately limit the interactions of iron and oxygen to only those that benefit the organism. Similarly, most iron not bound within heme is sequestered and buffered by intracellular storage and transport systems comprising proteins such as ferritin and ferroportin, thus mitigating iron-induced oxidative damage.

The careful dance of maintaining appropriate heme levels while limiting excess free iron encompasses many carefully regulated processes under the control of the NRF2 transcription factor. As highlighted in this review, NRF2 promotes the transcription of genes involved in both the synthesis (*e.g.*, *ABCB6* and *FECH*) and degradation (*e.g.*, *HMOX-1* and *BLVRB*) of heme. However, *in vivo* validation of the role of NRF2 in physiological heme and iron homeostasis phenotypes remains an important knowledge gap in the field.

Interestingly, iron treatment alone (100 μ*M* ferric citrate) cannot activate NRF2 (unpublished data). However, NRF2 mediates transcription of ferritin and ferroportin iron buffering systems (Table 1), which results in a reduced labile iron pool. Since the labile iron pool contributes to many cellular processes, a reduced labile iron pool from NRF2 activation is expected to produce a cellular phenotype. Consequently, the physiological impacts of NRF2 on iron metabolism are likely to initiate from NRF2 activation rather than from alterations to iron-mediated oxidative stress. It is also important to note that a certain species of ROS such as H_2_O_2_ also serves as an important signaling molecule (159). Emerging evidence showed that NRF2 also regulates the production of such signaling molecules through transcriptional modulation of heme-containing NAPDH oxidases (NOX) (69).

**Table 1.** Iron-Related Human Genes Regulated by NRF2

*Function*	*Symbol*	*Gene name*
Heme synthesis and O_2_ transport	*HBB*	Hemoglobin subunit beta
	*HBG1*	Hemoglobin subunit gamma 1
	*ABCB6*	ATP binding cassette subfamily B member 6
	*FECH*	Ferrochelatase
Heme catabolism	*HRG1/SLC48A1*	Heme-responsive gene 1
	*HMOX-1*	Heme oxygenase 1
	*BLVRA*	Biliverdin reductase A
	*BLVRB*	Biliverdin reductase B
	*AMBP*→A1M	Alpha-1-microglobulin/bikunin precursor→Alpha-1-microglobulin
Nonheme iron buffering	*FTH1*	Ferritin heavy chain
	*FTL*	Ferritin light chain
	*FPN1*	Ferroportin
	*PIR*	Pirin
Cancer progression	*HMOX-1*	Heme oxygenase 1
	*FTH1*	Ferritin heavy chain
	*FTL*	Ferritin light chain
Ferroptosis	*GPX4*	Glutathione peroxidase 4
	*SLC7A11*	Solute carrier family 7 member 11/System xCT
	*HMOX-1*	Heme oxygenase 1
	*FTH1*	Ferritin heavy chain
	*FTL*	Ferritin light chain
	*MT-1G*	Metallothionein-1G

The importance of NRF2 in mediating interactions between iron and oxygen is becoming apparent as we identify how deregulated NRF2 facilitates iron-mediated cellular pathologies: disruptions to HMOX-1 and ferritin signaling enable cancer progression and reduce treatment efficacies. Since NRF2 can alter iron signaling to facilitate cancer and inhibit treatment, it remains critical that we understand relationships between NRF2 and iron. Indeed, considering the GOE coincided with both the onset of biological utilization of iron and NRF2 evolution, understanding whether NRF2 was eventually selected as a coping strategy for the iron-mediated oxidative toxicity that accompanies iron-facilitated biology presents an intriguing question to entertain as novel relationships among NRF2, iron, and oxygen are uncovered.

## Abbreviations Used

A1M: alpha-1-microglobulin
ABCB6: ATP binding cassette subfamily B member 6
AMBP: alpha-1-microglobulin/bikunin precursor
ARE: antioxidant response element
BACH1: BRCA-1-associated carboxy-terminal helicase
BLVRA: biliverdin reductase A
BLVRB: biliverdin reductase B
ChIP: chromatin immunoprecipitation
EGCG: epigallocatechin 3-gallate
FECH: ferrochelatase
*FH*: fumarate hydratase
FIN: ferroptosis-inducing
FOXM1: Forkhead Box M1 transcription factor
FPN1: ferroportin
FTH1: ferritin heavy chain
FTL: ferritin light chain
GCLM: glutamate–cysteine ligase modifier subunit
GOE: Great Oxygenation Event
GPX: glutathione peroxidase
GPX4: glutathione peroxidase 4
GSH: monomeric, reduced glutathione
HIF1α: hypoxia-inducible factor 1α
HLRCC: hereditary leiomyomatosis and renal cell cancer
HMOX-1: heme oxygenase 1
HPSC: hematopoietic stem cell
HRG1: heme-responsive gene 1
IRE: iron response element
IRP: iron regulatory protein
KEAP1: Kelch-like ECH-associated protein 1
MT-1G: metallothionein-1G
NF-E2: nuclear factor erythroid 2
NF-κB: nuclear factor kappa-light-chain-enhancer of activated B cells
NRF2: nuclear factor (erythroid-derived 2)-like 2
PIR: pirin
ROS: reactive oxygen species
SLC7A11: solute carrier family 7 member 11/System xCT
StRE: stress response element
tBHQ: tert-butylhydroquinone
UTR: untranslated region
